# Diabetes management in Thailand: a literature review of the burden, costs, and outcomes

**DOI:** 10.1186/1744-8603-9-11

**Published:** 2013-03-14

**Authors:** Chaicharn Deerochanawong, Alessandra Ferrario

**Affiliations:** 1 Rajavithi hospital, College of Medicine, Rangsit University, Ministry of Public Health, Bangkok, 10400, Thailand; 2 LSE Health, London School of Economics and Political Science, Houghton Street, London, WC2A 2AE, UK

## Abstract

Management of diabetes represents an enormous challenge for health systems at every level of development. The latter are tested for their ability to continuously deliver high quality care to patients from the day they are diagnosed throughout their life. In this study, we review the status of diabetes management in Thailand and try to identify the key challenges the country needs to address to reduce the current (and future) medical and economic burden caused by the disease.

We conducted a literature review on the burden, costs, and outcomes of diabetes in Thailand. This information was complemented by personal communication with senior officials in the Thai Ministry of Health.

We identified the following priorities for the future management of diabetes in Thailand. First, increasing screening of diabetes in high risk population and promoting annual screening of diabetes complications in all diabetic patients. Second, identifying and addressing factors affecting poor treatment outcomes. Third, policy should specify clear targets and provide and use a monitoring framework to track progress. Fourth, efforts are needed to further improve data availability. Up-to-date data on the medical and economic burden of diabetes representative at the national level and at least the regional level are essential to identify needs and monitor progress towards established targets. Fifth, promotion of a healthy lifestyle for prevention of diabetes through education and quality information delivered to the public.

## Background

Thailand is an upper-middle income country in South-East Asia which has achieved impressive economic, social, and health improvements in the past ten years. Between 2007 and 2011 its gross domestic product grew by 7.8%, the percentage of people living below poverty line decreased from 21% to 8.1% between 2000 and 2009, and under-five mortality reached a record low for a country with 13 deaths per 1000 live births in 2010 [1].

The country’s disease burden and demographic profile stand out from the South East Asia region’s average for its high prevalence of non-communicable diseases (NCDs), higher than the average regional prevalence of HIV/AIDS, and an ageing population [2].

Non-communicable diseases are estimated to account for 71% of all deaths with cardiovascular diseases representing 27%, cancers 12%, and diabetes 6% of all deaths [3]. Communicable diseases are estimated to account for 24% of the total years of life lost (regional average 49%) while non-communicable diseases account for 55% (regional average 36%) [3].

Life expectancy in 2009 was 70 years (66 for males and 74 for females) [4]. The total fertility rate has decreased from 2.4 in 1990 to 1.6 in 2006 [5]. In terms of age structure (based on 2009 figures), 22% of the Thai population was below age 15 and 11% above age 60, the population median age was 33.

Many factors have contributed to these remarkable health achievements and among these, increased access to health care through universal health insurance is probably one of the key ones. Thailand was the first country in the region able to achieve universal health coverage of its population. Universal health coverage was achieved in 2002 after 27 years of progressive expansion of the universal coverage (UC) scheme to include all the uninsured together with the beneficiaries of the former medical welfare scheme for the poor, the elderly, the disabled and the children, and the voluntary health insurance scheme for the informal sector [6]. This scheme is financed through general taxation and covers 75% of the Thai population [7].

The two other main public schemes are the civil servants medical benefit scheme, financed through general tax and a non-contributory scheme, and the social security scheme which is financed by equal contributions from employer, employee and the government [8]. In 2010, these two schemes covered 9% and 16% of the population respectively [8].

In terms of the benefit package, the UC scheme covers for in-patient and out-patient care, prescription drugs, laboratory investigations, simple dental care procedures, disease prevention, health promotion, several expensive treatments such as radio- and chemotherapy, surgical procedures, and emergency treatment [7]. Since the end of 2008, renal replacement therapy including renal and peritoneal dialysis and kidney transplantation is also covered by the UC scheme although a co-payment per haemodialysis session is required [9].

Very little information is available on medicines availability in Thailand. According to a survey using the standard WHO/HAI methodology, in 2006 metformin was available in more than 80% of the surveyed public (20 hospitals) and private (21 private retail pharmacies) health facilities [10]. Diabetes medicine such as metformin, sulfonyurea and insulin are available to all diabetes patients free of charge as part of the UC scheme.

Management of diabetes represents an enormous challenge for health systems at every level of development. The latter are tested for their ability to continuously deliver high quality care to patients from the day they are diagnosed throughout their life. Achieving this requires good coordination across different areas of health care, different levels of care, in addition to trained human resources, an efficient supply system for medicines, a reliable health information system, national and international policies and strategies, and an equitable financing system which ensures access to essential health care services.

In this study, we review the status of diabetes management in Thailand and try to identify the key challenges the country needs to address to reduce the current (and future) medical and economic burden caused by the disease. To do that, we review evidence on the burden of diabetes, including the available data sources, available diagnostic and screening programmes, diabetes treatment, costs and outcomes, and policies implemented.

## Methodology

This study is based on secondary data analysis complemented by primary data collection. A literature review of peer-reviewed and grey literature including policy documents and annual reports of the MoPH and government statistics was undertaken. The following key words were used on the 21^st^ of February 2012 PubMed ((diabetes[Title]) AND Thailand[Title/Abstract]) OR (("Diabetes Mellitus"[Mesh] OR "Diabetes Mellitus, Type 2"[Mesh] OR "Diabetes Mellitus, Type 1"[Mesh]) AND "Thailand"[Mesh]). Studies presenting data on prevalence, incidence, mortality, outcomes (testing, screening rates), and complications were included. We excluded studies which did not present disaggregated data on diabetes (e.g. studies on chronic disease including diabetes). Evidence from the systematic literature review was complemented by primary data obtained through personal communication with Thai diabetes experts and senior public health officials from the Bureau of Non-Communicable Diseases (NCDs) and the Bureau of Policy and Strategy in the Thai Ministry of Public Health (MoPH).

## Results

The search strategy yielded 267 papers, of which 194 were excluded through title and 37 through abstract screening because they did not meet the inclusion criteria. There were 46 final peer-reviewed papers included in the review (Table 1).

**Table 1:** Literature review results

**Area of diabetes management**	**Number of references retrieved**	**References**
Prevalence and incidence	17	[11,12,16-25,27-29,33,34]
Mortality	2	[35,36]
Cost of diabetes	6	[37,38,52,60-62]
Prevalence of complications	15	[13,15,49-51,53,60,63-70]
Cost of complications	4	[38,52,60,61]
Outcomes	6	[50,53,63,67,71,72]

## Data sources on the burden of diabetes

### National health examination survey (NHES)

There is no national longitudinal diabetes registry in Thailand and the main source of prevalence data for diabetes is the national health examination survey (NHES). The aim of the NHES is to estimate the prevalence of particular health conditions and risk factors including obesity, diabetes, and mental, reproductive and elderly health. This survey was completed for the fourth time in 2009 (previous surveys were conducted in 1991, 1997, 2004). In 2009, a national representative sample of 20,450 (39,290 in 2009) individuals aged 15 and over was randomly selected using a four-stage sampling strategy from five provinces in each of the four regions and Bangkok. Diabetes prevalence was assessed by fasting blood glucose test and patients identified as diabetic if they had either FPG > = 7.0 mmol/L but lack of a previous diagnosis (undiagnosed diabetes) or a previous diagnosis of diabetes and intake of glucose lowering drugs during the past two weeks (diagnosed diabetes) [11,12].

### Diabetes registry project

In April 2003, 9,419 diabetes patients (both Type 1 and 2) from eleven tertiary care hospitals overall Thailand were enrolled in the Thailand diabetes registry (TDR) project [13]. This project was a collaboration between the Clinical Research Cooperation Network (CRCN) and the Health System Research Institute (HSRI), supported by the Endocrine Society of Thailand. The first objective of this registry was to identify the characteristics of Thai diabetic patients in tertiary care medical centers and to determine the extent of long term diabetic complications. The second objective was to develop and strengthen a clinical research network in Thailand which included experts in diabetes mellitus. The third and final objective was to collect baseline data for future follow-up studies. Cross-sectional data were collected from 11 tertiary level hospitals with diabetes clinics between April and December 2003. Demographic data, clinical status of diabetes and its complications were collected to estimate the prevalence of complications and risk factors. Data quality was ensured by regular on-site visits of internal and external auditors. This project also had a second component which was a three-year cohort study from April 2003 to February 2006 to determine the causes of death in diabetes patients.

### DiabCare Asia

DiabCare is an international collaboration between NovoNordisk Asia Pacific Pte Ltd, Singapore; BioRad Pacific, Hong Kong; and diabetes associations in the participating countries (Bangladesh, China, India, Indonesia, Malaysia, Philippines, Singapore, South Korea, Sri Lanka, Taiwan, Philippines, and Vietnam). The aim of this partnership is to collect evidence on the disease pattern, management, control status, and complications of diabetes in the Asian diabetes population. Patients were recruited in hospitals and followed for 8 to 9 months depending on the study year. DiabCare Asia studies were conducted in 1998 [14], 1991, 2001, 2003, 2008. The next data collection round is planned in late 2012- early 2013. Thailand was surveyed during the 1998, 2001 [15], 2003, and 2008 rounds.

### International collaborative study of cardiovascular disease in Asia (InterASIA)

The InterASIA study on diabetes prevalence, risk factors for cardiovascular diseases, and diabetes management was conducted in 2000 in Thailand and China. This study, founded by Pfizer was a collaboration between universities in Australia, China, Thailand, and the US. The study was based on a nationally representative sample of the Thai general population [16].

### Other studies

Several other studies have been conducted on the burden of diabetes in Thailand. These include investigations on the incidence [17,18] and prevalence of diabetes in population sub-groups [19], prevalence [20,21] and incidence [22-26] of diabetes Type 1 in children, incidence [27] and prevalence [28-30] of gestational diabetes, and prevalence of type 2 diabetes in women with polycystic ovary syndrome [31]. Other studies looked at the prevalence of complications in the Thai diabetes population.

## Prevalence and incidence

### Prevalence of diabetes mellitus type 2

**Figure 1:**
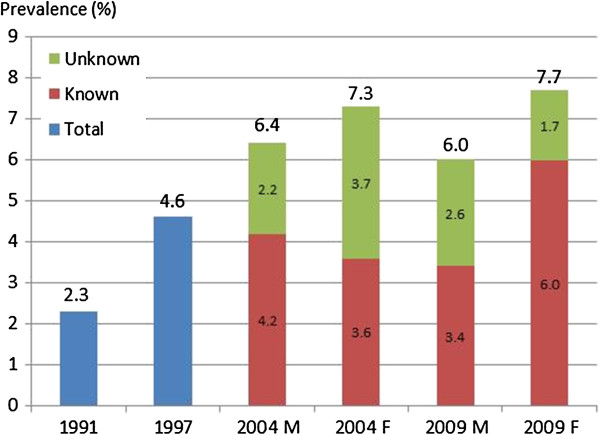
**Prevalence of diabetes mellitus in Thailand, 1991-2009.** Source: 1991, 1997, 2004, 2009 NHES I-IV. Notes: M: males, F: females. All estimates refer to people aged 15 and over. Diabetes was defined as FPG ≥ 126 mg/dl or previous diagnosis of diabetes and use of medication during the past two weeks.

Data from the four NHES indicate that prevalence of diabetes in individuals aged 15 and over has increased over time from 2.3% in 1991 to 4.6% in 1997, to 6.8% in 2004, and to 6.9% in 2009 [32] (Figure 1). According to NHES 2009 and 2004, women experienced a higher prevalence than men [11,12]. In contrast, findings from the InterASIA study in 2000 did not identify any difference in diabetes prevalence between men and women aged 35 and over (9.3% men vs. 9.9% women, p = 0.6) [16]. At the other end, an earlier study^a^ among Shinawatra employees, a group of relatively young individuals with high socioeconomic status, found a higher prevalence in men (2.2%, N = 1,250) than in women (0.1%, N = 2,365) [19].

According to the NHES 2009, prevalence of diabetes was higher in urban areas in comparison to rural areas (P < 0.001 for both sexes) [11]. However, findings from NHES 2004, only found a statistically significant higher prevalence in urban men, in comparison to their rural counterparts, (P < 0.001) but not in urban women (p > 0.05) [12]. The existence of a difference in diabetes prevalence between urban and rural areas in Thailand was confirmed by findings from the InterASIA study in 2000 (12.1% urban vs. 8.4% rural, p = 0.01, in individuals aged 35 and over) [16]. No difference between urban and rural areas was identified in the InterASIA study in 2000 and the NHES survey in 2004 [12,16].

Different studies on diabetes prevalence in Thailand agree that the prevalence of diabetes increases with age and reaches a peak at some point after age 55 depending on the study [11,12,16,19].

An important problem in diabetes care is under-diagnosis as it delays start of treatment and exposes the patient to the risk of complications which leads to higher treatment costs. Levels of under-diagnosis improved between 2004 and 2009 and this improvement was more evident in women than men. Nevertheless, a large proportion of total diabetes patients remains undiagnosed (from 66.5% to 47.3% in men and from 51.4% to 23.4% in women between 2004 and 2009) [11].

Findings from the NHES 2009 suggest that women have better rates of diagnosis in comparison to men and this difference was statistically significant (there was a difference also in 2004 but this was not statistically significant) [11,12]. However, the InterASIA study did not find a statistically significant difference in the proportion of diagnosed patient by gender (53% women vs. 47% men, p = 0.4) but it found that diagnosis rate was higher for people aged 55 and over (63% > =55 years old vs. 37% <54 years old, p = 0.01) [16].

A comparison of the findings from the NHES in 2009 with results from 2004, shows that the proportion of individuals with diabetes and concomitant hypertension did not significantly decrease in 2009 in both sexes [11]. However, the proportion of women with diabetes who were either abdominally obese or had high total cholesterol (≥5.2 mmol/L) increased from 18% in 2004 to 23.5% in 2009 and this difference was significant (both P < 0.01) [11].

An earlier study on the prevalence of DMT2 in children and adolescents (mean age 11.6 years) reported an increase from 5.8% to 13.3% between 1986 and 1995 [21]. The authors suggest a link between this increase and the concomitant increase in obesity from 5.8% to 13.3% between 1990 and 1996 [21].

### Incidence of diabetes mellitus type 1 in children

**Figure 2:**
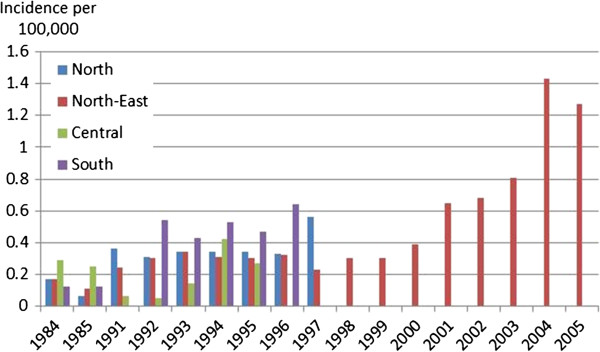
**Incidence of diabetes mellitus type 1 in children aged 0-15.** Sources [22-26]:. Secondary results from Tuchinda et al. 2002. Notes: Results from a study in Bangkok were not included as they were not comparable with the other studies due to the different methodology employed [24]. There was another study looking at the seasonal variation in DMT1 which was not included because it did not report incidence rates but only total number of cases in one hospital which made it unsuitable to calculate population incidence rates [20]. Incidence was calculated by dividing the total number of cases reported by hospitals with the total child population in the hospitals’ catchment area. Data were obtained from retrospective postal survey or medical record analysis and no information on the diagnostic criteria used was provided. A study summarising the results of the regional studies mentions that the criteria in the 1997 *Report of the Expert Committee on the Diagnosis and Classification of Diabetes Mellitus* were used.

Incidence of type 1 diabetes in children in North-East Thailand has increased over the years from in 0.17 per 100,000 in 1984 to 0.3 in 1995, to 0.39 in 2000 and 1.27 in 2005. In the other regions it has also grown although following a less linear trend particularly in the Central region (Figure 2).

Findings from a study in the Bangkok-Noi district in Bangkok, found an extremely small number of cases (maximum 1 per year) between 1991 and 1995 which, despite the size of the child population in this district, it still resulted in the highest prevalence rates for type 1 diabetes recorded in the country (2.18 per 100,000 in 1991, 0 in 1992, 1.97 in 1993, 2.06 in 1994, and 2.04 in 1995) [24].

Another study looked at the seasonal variation of diabetes type 1 and found that the peak seasons were the winter and the summer season and lower during the rainy season [20]. This was explained by the higher prevalence of infections in the winter season and the higher levels of pollution in the summer in comparison to the rainy season [20].

All studies (apart from the Bangkok study which did not discuss gender differences) on diabetes type 1 identified higher incidence in girls, with a girls to boys ratio ranging from 1.3 to 2. However, none of them tested for statistical significance. Peak age at onset was 10-14 in North and North-East region [22,25,26], 11-15 in the South region [23], and 9-12 in Bangkok [24].

### Incidence of diabetes mellitus type 2 in adults

We identified three studies on incidence of DMT2 in adults in urban Thailand from a high socio-economic background [17,18,33]. All the three studies used the diagnostic criteria from the American Diabetes Association (ADA) using fasting plasma glucose tests (FPGs), in addition to that one study also used oral glucose tolerance tests (OGTTs). The most recent study was among professionals and office workers in Bangkok and found an incidence rate in the age-group 35-60, of 17.8 per 1000 person-years (PY) in men and 9.2 per 1000 PY in women in 2005 [17]. A second study among university hospital employees in Bangkok reported an incidence of 13.6 per 1000 PY in men and 6.4 per 1000 PY in women between 2001 and 2005. The study participants were over 35 years old and predominantly female (three-quarters). High BMI (>25 kg/m2), elevated FPG (> = 96 mg/dl) and alanine aminotransferase levels (> 18 mg/dl) were shown to be independent predictors of DMT2 [18]. Risk of diabetes in men was approximately twice as high as in women but this apparent association was confounded by higher BMI and FPG levels in men and the crude rates showed there was no association between gender and DMT2 [18]. Findings from this study were supported by an earlier study which found an overall incidence of 11.3 per 1000 PY among employees of a state enterprise in Bangkok between 1985 and 1997 [33].

### Gestational diabetes

**Figure 3:**
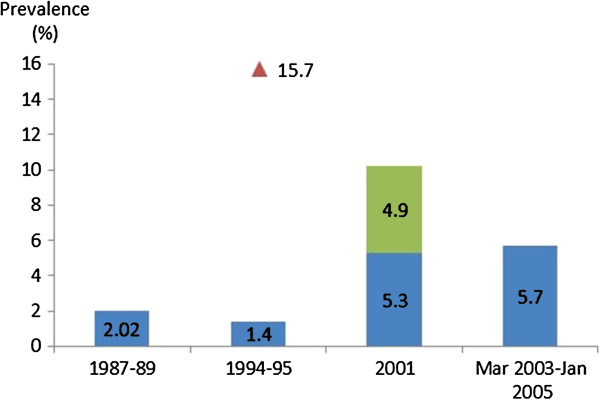
**Prevalence of gestational diabetes.** Notes: 1987-1989: gestation week not specified, NDDG criteria; 1994-95: 24-28 week of gestation, 1.4% NDDG criteria, 15.7% WHO criteria; 2001: 5.3% before 20 week of gestation, NDDG criteria; 2001: 4.9% additional at 28-32 weeks of gestation, NDDG criteria; March 2003-January 2005: mean gestational age 26.8 weeks, NDDG criteria, only women aged 30-34.

Four studies on the prevalence of gestational diabetes mellitus (GDM) were identified [27-29,34]. They were hospital based (one hospital per study) and mostly used the diagnostic criteria of the National Diabetes Data Group (NDDG), (50 g + 100 g OGTT) with one study also comparing NDDG results with WHO criteria (75 g OGTT). All hospitals were located in Bangkok (Figure 3).

The highest prevalence (15.7%) was estimated in 1995 for women in their 24^th^ to 28^th^ week of gestation using WHO criteria [34]. When using NDDG criteria, the same study estimated a remarkably lower prevalence of 1.4% [34]. Another study highlighted the importance of the timing of diagnosis, 5.3% of screened pregnant women were diagnosed with gestational diabetes before their 20^th^ gestational week and an additional 4.9% of previously undiagnosed women were identified in a second round of testing during their 28^th^ to 32^nd^ week of pregnancy [27]. The 1987-1989 study followed a sub-sample of women after delivery by performing an OGTT using WHO criteria 4-6 weeks after childbirth. Results showed that 42.2% of the 71 women tested had abnormal carbohydrate tolerance, 7% had diabetes and 35.2% impaired glucose tolerance (IFG) [28].

The most recent study (March 2003-January 2005) aimed to assess the percentage of pregnancies with GDM which were missed at the time the study was conducted because clinical guidelines in the study hospital limit screening to women at high risk of developing GDM. Eligibility criteria for screening included pregnant women with at least one of the following risk-factors: aged 35 and over, family history of diabetes, previous birth over 4 kg, and other known risk-factors of GDM. This study showed that the prevalence of women aged 30 to 34 was 5.7%, and that of the 32 cases identified, 12 had one risk factor, one had two risk factors (1). More importantly 18 cases of GDM showed no risk factors and would have been missed due to the recommendations in the hospital’s guidelines at the time of the study [29].

## Mortality

Results of a comprehensive study to verify causes of death using medical records and verbal autopsy methods, suggest that the leading cause of death in both men and women in 2005 was stroke (9.4% and 11.3% respectively) [35]. Diabetes was the second cause of death in women (8.0%) and the tenth cause of death in men (3.2%) in 2005 [35]. This difference is partly explained by the high mortality levels due to road accidents and HIV/AIDS in men. This study also highlighted issues with vital registration where a high percentage of deaths were classified as ill-defined. Adjustments to vital registration data led to important changes in the proportion of deaths due to HIV/AIDS, ischaemic heart disease, and diabetes [35]. The proportion of deaths attributable to diabetes reaches its maximum at age 50-79. In 2005, they represented the main cause of death in females (12.3%) and seventh in males (5.4%) in this age group [36].

## Costs

Using a micro-costing approach, a study including 475 patients receiving treatment in a hospital in North-East Thailand estimated a median cost of illness per patient of USD 140 (mean USD 881) in 2008 (1 USD = 32 THB, at 2008 prices) [37]. The total annual cost included 23% direct medical costs, 40% direct non-medical costs, and 37% indirect costs [37]. Direct medical costs were driven by inpatient service costs (11% of total cost of illness), while direct non-medical cost were dominated by the cost of informal care (28% of total cost of illness). Cost of permanent disability (19% of total cost of illness) was the most important contributor to informal cost [37]. The median cost of illness increased with age (p-value < 0.001), duration of the disease (p-value < 0.001), the level of fasting blood glucose level (p-value = 0.002) and number of co-morbidities (p-value = 0.0013) [37]. The median cost of DMT1 was higher than DMT2 (USD 748 vs. USD 140), however this difference was not statistically significant (p = 0.167 [37]).

The monthly median cost of informal care based on a sample of 190 informal care givers was estimated at USD$ 27 using an opportunity cost approach and USD$ 23 using the proxy good method [38]. Cost of informal care contributed 28% of total cost of illness of diabetes [37] (Table 2).

**Table 2:** Direct and indirect cost of diabetes in Thailand

**Estimate**	**Summary of the main features of the study**	**Reference**
**Direct and indirect costs**		
Median and (mean) cost of illness per type of patient per year	Study year: Oct 2007- Sep 2008	[60] same study as [37,38]
Independent: USD$ 124 (USD$ 598), SD 2152	Reference year for estimate is the fiscal year 2008	
Disabled: USD$ 811 (USD$ 2,700), SD 4982	Setting: Waritchaphum Hospital. A 30-bed public district hospital in Sakhon Nakhon province in northeastern Thailand	
	Sample and study design: 475 randomly selected diabetic patients. Prevalence-based cost of illness, societal perspective	
Median and (mean) cost of illness per patient (both disabled and independent) per year: USD 140 (USD$ 881),[82.01-552.50]	Study year: Oct 2007- Sep 2008	[37] same study as [38,60]
This included 23% of direct medical cost, 40% of direct non-medical cost, and 37% of indirect cost	Reference year for estimate is the fiscal year 2008	
Informal care contributed to 28% of total cost of illness	Setting: Waritchaphum Hospital. A 30-bed public district hospital in Sakhon Nakhon province in northeastern Thailand.	
	Sample and study design: 475 randomly selected diabetic patients. Prevalence-based cost of illness, societal perspective	
**Direct costs**		
Average public treatment cost per patient per year was USD 95	Study year: Oct 2007- Sep 2008	[62]
Drug cost was the highest cost component (25% of total cost), followed by inpatient cost (24%) and outpatient visit cost (17%).	Reference year for estimate is the fiscal year 2008	
	Setting: Waritchaphum Hospital. A 30-bed public district hospital in Sakhon Nakhon province in northeastern Thailand.	
	Sample and study design: 475 randomly selected diabetic patients. Retrospective prevalence-based cost of illness study, provider perspective	
Annual average cost of illness (including patients with complications): USD$ 158 (THB 6,331)	Study year: October 2000-September 2001	[52]
Contribution to the total cost: 45% pharmacy services, 24% outpatient services, 16% inpatient services, 11% laboratory investigations.	Setting: 30-bed public community hospital in central Thailand	
Annual cost for DMT2 and DMT1 patients with no complication USD$ 101 (THB 4,037) and USD$ 251 (THB 10,059) respectively	Sample and study design: 186 diabetes patients. Retrospective prevalence-based cost of illness study, provider perspective	
**Indirect costs**		
Median and (mean) cost of informal care per month	Study year: 2008	[38] same study as
Opportunity cost approach: USD 27 (USD$ 37)	Setting: Waritchaphum Hospital. A 30-bed public district hospital in Sakhon Nakhon province in northeastern Thailand.	
Proxy good method:	Sample and study design: 190 informal caregivers. Interview with carers, revealed preference method	
USD$ 23 (USD 34)		
Average time spent on informal care was 112 hours per month		

SD: Standard deviation, [] interquartile range.

Findings from one hospital showed that the cost of informal care accounted for 28% of the total cost of diabetes [38]. This is likely to have negative repercussions on labour force participation of informal carers who dedicated on average 112 hours per month to informal care and were in their most productive age (25-54 years old) [38].

## Diabetes screening and prevention

Recently, Thailand launched the new “Thailand Healthy Lifestyle Strategy 2011-2020 Plan” endorsed by the Ministry of Public Health [39]. This plan aims to decrease the prevalence, complications, disability, mortality and cost of illness of five major non-communicable diseases including diabetes, hypertension, ischemic heart disease, stroke and cancer. The strategies proposed include: healthy public policy, social mobilization and public communication, community building, surveillance and care system including screening diabetes in high risk population, and capacity building [39].

Yet, to-date, there is still no national screening and no prevention programme in place. There are some examples of successful sub-national diabetes screening and prevention initiatives. These include a prevention model for diabetes in primary care, an educational programme on diabetes prevention for community health care workers, and use of a mobile health unit to screen people living in rural areas for chronic and other health conditions [40-44]. Another example is the development of a simple diabetes risk score to identify Thai patients at high risk of developing diabetes. This score is based on a set of variables (age, BMI, waist circumference, hypertension, and history of diabetes in parents or siblings) which were identified as being significant predictors of diabetes [33]. Measurement of these variables does not require laboratory tests, making the model a cost-effective instrument to identify high risk people to be screened.

However, despite these initiatives have shown initial positive results, they not really been taken beyond the initial pilot phase.

## Diabetes treatment

Thailand has its own guidelines for diabetes treatment (only available in Thai) [45] endorsed by the Thai Diabetes Association, the Endocrine Society of Thailand, and the Ministry of Public Health. The guidelines are updated every three to four years and the latest version was published in 2011. More than 5,000 copies have been published and distributed to primary and secondary care physicians in all the regions of Thailand and they are also available online. However, there is no evidence on how many physicians have adopted these guidelines.

The Thai guidelines resemble the WHO/IDF guidelines [46,47], particularly with reference to screening, prevention, treatment and monitoring. However, use of HbA1c is not universally recommended for the diagnosis of diabetes because of the lack of standardization of the HbA1c measurement methodology in Thailand and FPG is recommended instead. HbA1c is still generally recommended for monitoring treatment outcomes.

To control diabetes and reduce the risk of developing complications, it is essential to optimise physiological values such as glycaemic levels and blood pressure and to perform regular examinations for nephropathy (albumin excretion, serum creatine), retinopathy (eye), neuropathy (distal symmetric polyneuropathy), and foot disease. Evidence suggests that the frequency with which these tests are performed in Thailand is suboptimal. A study in urban primary care clinics found that annual eye and foot examination was performed only in 21.5% and 45% of patients, respectively [15] while the ADA recommends annual testing starting at diagnosis [48].

Another study in the outpatient department of a university hospital found that the annual eye, urine albumin excretion, serum creatinine, and foot examination was performed in 38.4%, 42%, 83.5%, and 17.3% of patients in 2006 [49].

The highest rate for DMT2 patients receiving retinal examination, 75.6% between April to December 2003, was found in the diabetes registry project which included tertiary diabetes clinics [50].

## Diabetes complications and cost of complications

Overall, prevalence of diabetic retinopathy (DR) ranged between 13.6-31.2% and it mostly involved non-proliferative DR while the prevalence of diabetes nephropathy ranged between 24-43.8% and was higher in patients with concomitant DR (Table 3). A variety of vascular complications ranging from absence of foot pulse to ulcer and gangrene leading to foot amputation and stroke were reported.

**Table 3:** Prevalence of diabetes complications in Thailand

**Estimate**	**Summary of the main features of the study**	**Reference**
**Prevalence of diabetic retinopathy (DR)**		
15.1% DR, 11.6% non-proliferative, 3.5% proliferative DR	Study year: March-October 2007	[53]
	Setting: 13 primary care units in urban areas	
	Sample and study design: Cross-sectional study, ADA criteria	
	287 diabetic patients (79 males, 208 females)	
31.2% (n = 86), 25% (n = 69) non proliferative and 6.2% (n = 17) proliferative DR	Study year: 1 Jan-31 Dec 2006	[49]
	Setting:1 tertiary hospital, out-patient department	
	Sample and study design: 722 diabetes patients. Retrospective records review of DMT2 patients	
31.4% (n = 2105), 22% (n = 1475) non-proliferative and 9.4% (n = 630) proliferative DR	Study year: April-December 2003	[50]
	Setting: 11 tertiary diabetes centres	
	Sample and study design: 6,707 diabetes Type 2 patients (4,434 females, 2,273 males). Cross-sectional, hospital based study. Thailand diabetes registry project	
DMT2 and DMT1	Study year: April-December 2003	[13]
30.7% (n = 2187), 21.3% (n-1516) non-proliferative,9.4% (n = 671) proliferative	Setting: 11 tertiary care medical centres	
	Sample and study design: 9,419 diabetes patients, 65.9% females, 94.6% DMT2, 4.5% DMT1, 0.1% MODY. Cross-sectional, hospital based study. Thailand diabetes registry project	
DMT1	Study year: April-December 2003	[63]
21.6% (n = 75), 10.9% (n = 38) non-proliferative and 10.7% (n = 27) proliferative DR	Setting: 11 tertiary diabetes centres	
	Sample and study design: 347 diabetes Type 1 patients (215 females, 132 males). Cross-sectional study of diabetes patients in 11 hospitals. Thailand diabetes registry project.	
22% (n = 667), 19% (n = 576) non-proliferative DR, 3% (n = 91) proliferative DR	Study year: January-December 2002	[65]
	Setting: All community hospital in Lampang province	
	Sample and study design: 3,049 diabetes patients (838 males, 2,211 females). Cross-sectional study in hospitals	
13.6% DR	Study year: 2001	[15]
	Setting: 8 provincial hospitals plus 4-5 district hospitals providing primary health care services in each province. Total number of sites: 37	
	Sample and study design: 1,078 diabetes patients (300 males, 778 females). Cross-sectional study	
Comprehensive eye examination:	Study year: NA	[69]
19.2% non-proliferative, 1.1% proliferative for the right eye, 18.5% non-proliferative, 1.3% proliferative for the left eye	Setting: Trang provincial hospital	
Photography:	Sample and study design: 714 diabetes patients. Cross-sectional, hospital based study	
23.8% NDR, 1.4% PDR for the right eye, 22.6% NDR, 1.3% PDR for the left eye		
**Diabetic nephropathy (DN)**		
DN without DR	Study year: January 2007-September 2008	[68]
62.8% normo-albuminuria, 26% micro-albuminuria, 11.2% macro-albuminuria	Setting: 7 public hospitals: Bangkok (3), Nakhonpathom (1), Prathumthani (1), Prathumthani (1), and Prathumthani (1)	
DN and DR	Sample and study design: 877 patients with diabetes Type 2 (ADA criteria), collection of urine samples. Cross-sectional study in the out-patient department of seven public hospitals	
18.5% normo-albuminuria, 35.5% micro-albuminuria, 48% macro-albuminuria		
Microalbuminuria 28.7%, macroalbuminuria 5.7%, 85% of them were non-DR and 15% DN and DR including 8% with both DN and DR	Study year: March-October 2007	[53]
	Setting: 13 primary care units in urban areas	
	Sample and study design: 287 diabetes patients, 79 males, 208 females. Cross-sectional study, ADA criteria	
Prevalence of DN 48.3% (Type 1) and 31.8% (Type 2)	Study year: April-December 2003	[67]
	Setting: 11 tertiary diabetes centres	
	Sample and study design: Children and adolescents diabetes, 58 Type 1 and 22 Type 2, were screened for nephropathy. Cross-sectional study. Thailand diabetes registry project	
Prevalence of DN was 42.9%: 19.7% micro-albuminuria, 23.2% overt nephropathy.	Study year: April-December 2003	[73]
	Setting: 11 tertiary centres	
	Sample and study design: 4,875 diabetes patients, 63.8% females. Cross-sectional study, hospital based study. Thailand diabetes registry project	
Prevalence of DN 17%	Study year: 2001	[15]
	Setting: 8 provincial hospitals plus 4-5 district hospitals providing primary health care services in each province. Total number of sites: 37	
	Sample and study design: 1,078 diabetes patients (300 males, 778 females). Cross-sectional study	
**Vascular complications**		
Previous history of any lower extremity amputation: 0.9% right foot lesion, 0.6% left foot lesion	Study year: Aug 2005-March 2007	[74]
Diminished or absent pedal pulses 7.4% right foot lesion, 7.7% left foot lesion	Setting: Diabetic clinic in a university hospital in Northern Thailand	
Low-risk ulcerative foot 3.7%	Sample and study design: 438 diabetic patients. Baseline data of patients attending the clinic were collected	
High-risk ulcerative foot 0.2%		
1.2% acute foot ulcer/gangrene, 6.9% healed foot ulcer	Study year: 2001	[15]
1.9% stroke, 0.7% myocardial infarction	Setting: 8 provincial hospitals plus 4-5 district hospitals providing primary health care services in each province. Total number of sites: 37	
	Sample and study design: 1,078 diabetes patients (300 males, 778 females). Cross-sectional study	
Diabetic foot 40% (n = 50), cardiovascular disease 28.9% (n = 201), cerebrovascular disease 10.6% (n = 74)	Study year: 1 Jan-31 Dec 2006	[49]
	Sample and study design: 722 diabetes patients, Retrospective review of medicinal records of DMT2 patients	
DM duration more than 15 years vs. DM duration less than 15 years group	Study year: April-December 2003	[66]
DN 49.4% vs. 33.9%, DR 54.3% vs. 22.8%; myocardial infarction 9.4% vs. 3.5%, peripheral arterial disease, 17.3% vs. 5.5%, foot ulcer 13.4% vs. 5.3%,, stroke 9.4% vs. 7.0% and amputation 5.5% vs. 2.0%; all p values less than 0.01).	Setting: 11 tertiary diabetes centres	
	Sample and study design: 9,284 adult diabetes Type 2 patients registered to the Diabetes registry project, 2,244 patients with duration of diabetes more than 15 years and 7,040 patients with duration of diabetes less than 15 years. The longer duration group was on average older than the shoter duration group. Cross-sectional study. Thailand diabetes registry project	
Ischaemic heart disease 8.1% (n = 761)	Study year: April-December 2003	[13]
Cerebrovascular 4.4% (n = 415)	Setting: 11 tertiary care medical centres	
Peripheral vascular disease 11.4% (including amputation 1.6%, foot ulcer, and absence of peripheral pulse)	Sample and study design: 9,419 diabetic patients, 65.9% females, 94.6% DMT2, 4.5% DMT1, 0.1% MODY. Cross-sectional, hospital based study. Thailand diabetes registry project	
**Chronic kidney disease (CKD)**		
Prevalence of CKD stage 3 to 5 was 27.09% (n = 194) and 25.38% (n = 181) using Cockcroft-Gault formula and Modification of Diet in Renal Disease (MDRD) formula respectively	Study year: April- August 2007	[51]
	Setting: Six primary health care units in Udon Thani province	
	Sample and study design: 714 diabetic patients, 542 females and 174 males. Cross-sectional study, cluster random sampling method	
CKD stage 1 23.2% (n = 113), stage 2 28.7% (n = 140), stage 3 37.3 (n = 182), stage 4 8.2% (n = 40), stage 5 2.7% (n = 13)	Study year: 1 Jan-31 Dec 2006	[49]
	Setting: 1 tertiary hospital, out-patient department	
	Sample and study design: 722 diabetes patients. Retrospective review of medical records of DMT2 patients	
7.7% patients had renal insufficiency and 0.47% end-stage renal disease	Study year: April-December 2003	[73]
	Setting: 11 tertiary centres	
	Sample and study design: 4,875 patients, 63.8% females. Cross-sectional study, hospital based study.	

Two studies reported the prevalence of chronic kidney diseases (CKD). A study in a primary care diabetes centre reported a prevalence of 25-27 (depending on the estimation method) for CKD stage 3-5 in 2007 [51] while a second study in the outpatient department of a tertiary hospital reported a higher prevalence (37% stage 3, 8.2 stage 4, 2.7 stage 5) in 2006 (Table 3) [49].

Complications have a major impact on the cost of diabetes (Table 3). A study predicted the cost of diabetes to increase up to 232% depending on the type of complication [52].

The median cost of illness for patients with complications was significantly higher than for people without complications (USD$ 479.93 vs. USD$ 115.12, p < 0.001) and increased with increasing numbers of complications (p < 0.001) [38].

Disability was a major driver of diabetes cost as well as complications due to vascular problems leading to heart failure and corresponding surgery.

In terms of potential savings from better prevention, a study showed that preventing gangrene in DMT2 patients would generate almost USD$ 250 (THB 10,000) per patient per year [52].

## Diabetes outcomes

There are no national diabetes outcome indicators routinely collected in Thailand. Some diabetes centres report outcomes but this practice is voluntary and not standardised across the country.

The percentage of diabetes patients who were treated and controlled increased between 2004 and 2009 yet it remained low in addition to showing a large gender gap (men 7.7% in 2004 and 17.5% in 2009, women 15.8% in 2004 and 33.9% in 2009) [11]. Treatments rates for diagnosed patients were high leaving only 5.6% men and 1.9% diagnosed women without treatment [11]. However, 30% of treated men and 41% of treated women still did not attain diabetes control [11].

Diabetes control in patients with high blood pressure and high total cholesterol improved in 2009 from 3.4% to 12.2% in men and from 6.4% to 13.8% in women for hypertension between 2004 and 2009, 4% to 16.3% in men and 3.8% to 17.3% in women for high cholesterol between 2004 and 2009 [11]. However, at the same time the proportion of treated but not controlled patients increased from 25.4% to 36.3% in men and from 31.3% to 54.9% in women for hypertension and from 7.6% to 12.6% in men and from 7.0% to 18.5% in women for high cholesterol [11].

Evidence from tertiary care units in earlier studies (2003) shows a very high proportion of diabetes patients with poor glycaemic control (more than 70% of diabetes patients with HbA1c >7%) (Table 4). A more recent study (2007) in primary care units shows a slightly better figure (41.3% of patients with HbA1c levels <7%) (Table 4) [53].

**Table 4:** Control of diabetes and of HbA1c levels

**Estimate**	**Summary of the main features of the study**	**Reference**
HbA1c levels <7%	Study year: March-October 2007	[53]
41.3%	Setting: 13 primary care units in urban areas	
	Sample and study design: 287 diabetic patients, 79 males, 208 females. Cross-sectional study, ADA criteria	
HbA1c levels <7%	Study year: July 2007	[72]
29.7%	Setting: diabetes clinic in a community hospital	
	Sample and study design: 325 diabetic patients (who had diabetes for at least one year), 114 males, 221 females. Cross-sectional study.	
30.2% of the participants achieved HbA1c levels of less than 7%	Study year: April-December 2003	[71]
	Setting: 11 tertiary diabetes centres	
	Sample and study design: 8,913 Type 2 diabetes patients aged 18 and older (3,012 males and 5,901 females). Cross-sectional study. Thailand diabetes registry project	
HbA1c levels < 7%	Study year: April-December 2003	[67]
DMT1: 17%	Setting: 11 tertiary diabetes centres	
DMT2: 21.6%	Sample and study design: Children and adolescents diabetes, 58 Type 1 and 22 Type 2, Cross-sectional study. Thailand diabetes registry project	
HbA1c levels 7-8%		
DMT1: 20%		
DMT2: 15.2%		
HbA1c levels 8-9%		
DMT1: 15%		
DMT2: 15.2%		
HbA1c levels >9%		
DMT1: 47.6%		
DMT2: 48.2%		

Notes: standard deviation (SD).

## Access to treatment and inequalities

In principle, availability of medicines to treat diabetes in Thailand should be adequate since the universal health coverage policy covers more than 75% of the Thai population (the rest of the population is covered either by the civil servant or the social security scheme). Essential diabetes drugs such as metformin, sulfonylurea, pioglitazone, and insulin are included in the national drug list to which all UC insurees have access to. However, a study on diabetes mortality found that patients on the UC scheme were more likely to die than patients on the civil service scheme (adjusted hazard ratio 1.96, 95% CI 1.48-2.58, p-value < .005) [54]. Possible reasons for this include lower level of education and socioeconomic status, reduced access to lipid-lowering treatment (at the time of the study) and renal replacement among UC insured people in comparison to civil servants [54,55]. When the study was conducted between 2003 and 2006, low-cost generic statins were not yet available. This meant that many patients were not able to access treatment because of the high cost of the original drug. However, since the introduction of generic statins and their inclusion in the UC benefit package, this is no longer an issue.

Inequalities in access to treatment still persist in Thailand despite universal health coverage. Renal replacement therapy (RRT) for example was initially excluded from the benefit package of the UC scheme because the annual cost of haemodyalysis was four times higher (BHT 400,000, USD 12,100) than the price per quality-adjusted life year threshold set by the National Health Security Office (BHT 100,000, USD 3,0000) [9]. In 2008, thanks to pressure from patients and the public, renal replacement therapy was finally included in the UC benefit package. However, patients opting for haemodyialysis (which is more expensive than peritoneal dialysis) need to pay one third of the treatment cost out-of-pocket which corresponds to an annual cost of (BHT 133,333, USD$ 4,033) [9] in a country with a per capita gross domestic product of USD$ 4,608 in 2010 [1]. Further, geographical barriers can affect access to treatment and monitoring for patients living in remote areas.

## Policies

In 2004, the Ministry of Public Health launched the programme “Healthy Thailand” in an attempt to address the growing burden of NCDs. This programme aimed to promote a healthy lifestyle and to screen 60% of population aged over 40 years to be screened for IFG and diabetes by the end of 2006 [12]. However, due to lack of data, it is not clear whether this target was achieved.

Reduction of diabetes morbidity and mortality rates due to diabetes is one of the 17 targets included in the Health Thailand Strategy 2004-2015 [56]. However, this target does not include measurable objectives and a strategy to achieve this reduction.

Mobile eye care is the new project that headed by the Ministry of Public Health to improve the access to diabetes care with the aim of preventing blindness from diabetes retinopathy. People living in rural areas often face difficulties in accessing health care. They often have to travel long distances and the transportation costs can be unaffordable to many. One way of alleviating this is the use of mobile clinics which travel to remote areas and provide health care to where people live. The project will be initially implemented in 11 provinces in the northern and north-eastern of Thailand. With time, the plan is to expand the project to cover the entire country [57].

The Thailand healthy lifestyle strategic plan (2011-2020) lists reduction of diabetes incidence, complications, disability, mortality, and expense as one of its five main development goals together with reduction of hypertension, heart disease, cerebrovascular disease and cancer [39]. This should be achieved through the promotion of a balanced diet, adequate physical exercise, and suitable emotional management. However, none of the eighteen short- to long-term performance indicators is linked to measurable objectives in terms of disease burden and cost reduction. Instead they simply mandate for either a reduction or an increase of the relevant indicator. At a higher level, the Thai healthy life-style strategy aims to establish political will, raise public awareness, focus on preventive measures, seek wider collaboration and strengthening active engagement from all stakeholders including public and private sectors, civil society and the general public. Finally, it also seeks to institutionalise organizational structures at all levels of the society, from national to village level, to serve as implementation units responsible for coordination, policy direction, budget allocation, and monitoring and evaluation [39].

## Challenges in diabetes management according to Thai diabetes experts and senior public health officials

The strong features of the national policy and implementation framework for diabetes prevention and control in Thailand include the existence of capable staff and competent healthcare workers throughout Thailand, the presence of academic experts connected nationally and internationally, effective senior level management, strong policy development processes at the national level, and the experience of successfully implementing the tobacco control programme. Weaknesses include unorganised local and mid-level management, staff shortages, high workloads in rural areas, little time or opportunity for continuing training, and weak resource management.

Key opportunities would focus on a strong national policy response to diabetes and other NCDs, a strong network of competent health care workers and hospitals, budget support from both public and private sectors, and scientific and technical support from academics and researchers. The main threats identified are the negative influence of the media leading to changes in lifestyles and increasing risks for diabetes; inconsistent and unreliable information about diabetes disseminated to the public; low public awareness of diabetes issues especially among less well educated people; and high healthcare worker turnover in rural areas.

## Discussion

### Prevalence

The NHES is a nationally representative survey of the health status of the Thai population. NHES III and IV found a higher prevalence of DMT2 in women, older individuals, and in urban areas^b^. Under-diagnosis was higher in men (2009: 47.3 vs. 23.4%, respectively; *P* < 0.001) [11,12] and in those with less than secondary education [11]. Both surveys showed that nearly all patients who were diagnosed with diabetes were also treated with glucose-lowering medications (2004: men 2.6%, women 1.7%; 2009: men 5.6%, women 1.9% of diagnosed patients were treated) [11]. However, despite the high treatment rates, the percentage of treated and controlled patients (receiving treatment with glucose-lowering medication and with FPG <7.2 mmol/L) was still low (2004: men 7.7%, women 15.8%; 2009: men 17.5%, women 33.9% treated and controlled patients) [11].

The high percentage of treated patients among diagnosed diabetics suggests the availability of a resilient health care system in terms of access to treatment. However, access is hampered by the low diagnosis rates which need to be improved by increased screening of high-risk groups. A risk score to identify individuals at high-risk of developing diabetes was developed for the Thai population [33]. Wider implementation of this low-cost instrument can help identify high-risk individuals to be screened and therefore to increase the percentage of diabetes patients who is diagnosed. This would allow earlier start of treatment and could help in preventing part of the costs of complications arising from neglect of the disease. Another issue is the low rates of treated and controlled patients. To address these issues evidence is needed on factors responsible for poor treatment outcomes (e.g. patient compliance to treatment, performance of monitoring and self-management, etc.)

### Incidence of type 1 in children

Data on diabetes type 1 in Thai children aged 0-15 suggests an increased incidence over the last 20 years from less than 0.3 cases per 100,000 in 1984 in all regions to 1.27 cases per 100,000 in the North-East region in 2005. However, interpretation of this data requires caution. Apart from the Bangkok study, all the other studies restricted their data collection to hospitals. Incidence was calculated by dividing the total number of cases reported by hospitals with the total child population in the hospitals’ catchment area. Although hospitals’ response rate was generally high (range: 84.7% to 94.5%) [22,25], this means that in addition to not capturing diabetes type 1 cases who did not attend a hospital, the results were also importantly influenced by missing data from hospitals that did not participate in the survey.

Under-diagnosis is likely to have played a major role in the 1990s and first half of the 2000s because of incomplete insurance coverage which created barriers to health care access. Furthermore, apart from the North-East region, no data is available after 1997 and the most recent incidence data for the North-East region are for 2005.

### Incidence of type 2 diabetes in adults

The latest data on the incidence of DMT2 in Thai adults show an incidence rate of 13.6 in men per 1000 PY and 6.4 per 1000 PY in women [18]. However this data is out-of-date as it refers to the time period 2001 to 2005 and is not representative of the entire country as it based on urban high-socio-economic status individuals working in the healthcare sector.

### Gestational diabetes

Due to different gestational weeks and age of the prospective mother when the glucose test was conducted, it is not possible to draw definitive conclusions on the evolution of the prevalence of GDM over time. Data only included women attending antenatal care which might be biased towards higher socio-economic groups due to the lack of universal coverage at the time of the surveys. Despite these challenges, two main findings seem to emerge. First, a very large difference (eleven-fold) was reported when using different diagnostic criteria (NDDG (1.4%) vs. WHO criteria (15.7%)) on the same sample of women and another recent study that used the new criteria of international association of the diabetes and pregnancy study groups (IADPSG) found the prevalence of GDM in Bangkok was 23.0% [58]. Second, use of eligibility criteria which limit screening to women at high risk of developing GDM independently of their age have shown to miss more than 50% of cases among women aged 30 and over.

### Costs

Data on the cost of diabetes without complications mainly comes from a study in one-hospital in North-East Thailand. Generalisability of local studies on cost of illness is affected by variation of input prices across the country, the level of care of the hospital analysed, and the patient status. One study in Thailand for example showed that a visit to the regional hospital was 3.48 times more expensive than a visit to a community hospital (THB 1,181 vs. THB 339 in 2002) [59]. Another issue was the uncertainty and large variation around the results (very large standard deviations were reported).

### Complications and cost of complications

According to data from the Thailand diabetes registry, diabetic nephropathy was the most common complication accounting for 43.9% of all complications followed by retinopathy (30.7%), ischaemic heart disease (8.1%), and cerebrovascular disease (4.4%) [13]. Another study in the out-patient department of a university hospital found a lower prevalence for diabetic nephropathy, 37%, a similar prevalence for retinopathy, 31.2% but a substantially higher prevalence for cardiovascular and cerebrovascular disease, 28.9% and 10.6%, respectively.

The first study also highlighted the existence of high prevalence of risk factors for diabetes and its complications (dyslipidaemia, hypertension, and obesity (BMI ≥ 25 kg/m^2^) was 73.3%, 63.3%, and 52.6% respectively) [13].

Complications are the single largest driver of the cost of diabetes because they require more intensive care such as hospitalisation and often surgery. Studies showed that the median cost of illness for patients with complications in comparison to patients without complications was USD$ 480 vs. USD 115 [37], USD$ 190 for diabetic foot, USD$ 260 for cerebrovascular event, USD$ 336 for gangrene in comparison to USD$ 101 for patients without complications [52]. Interestingly, the latter study which was based on retrospective data analysis and cost forecast, found that the highest proportion of treatment cost was caused by pharmacy services (45%) followed by outpatient (24%) and inpatient services (16%) [52]. The first study found a similar share of inpatient costs (11%) but in addition to direct costs it also estimated indirect costs and the contribution of informal care, mortality, and permanent disability to the cost of illness (27.8%, 17.5%, 18.7%, respectively) [37] (Table 5). This data shed some light on substantial indirect costs caused by morbidity and reduced ability to work. Preventing complications, and related disability, by improving diabetes control is therefore of paramount importance to reduce the health and economic burden of diabetes.

**Table 5:** Cost of diabetes complications in Thailand

**Cost of complications**	**Summary of the main features of the study**	**References**
Median cost per year (USD$ at 2008 prices) of diabetes for patients:	Study year: 2007-2008	[60]
With complications: USD$ 480 (n = 148, SD = 3,023)	Setting: Waritchaphum hospital in Sakhon Nakhon province (North-East Thailand)	
Without complications: USD$ 115 (n = 327, SD = 2,648)	Sample and study design: 475 randomly selected diabetic patients	
Independent: USD$ 124 (n = 411, SD = 2,152)		
Any level of disability: USD$ 811 (n = 61, SD = 4,982)		
Mildly disabled: USD$ 668 (n = 51, SD = 3,848)		
Moderate disability: USD$ 2,374 (n = 7, SD = 7,940)		
Severely disabled: USD$ 4,891 (n = 1, SD = 0)		
Very severely disabled: USD$ 4,378 (n = 5, SD = 7,622)		
Average spending per hospital admission as percentage of GDP per capita and in USD (constant 2000):	Study year: 2006-2008	[61] USD equivalents of the percentage of GDP spent per hospital admission were calculated by the authors using GDP data (USD constant 2000, 2,712 in 2007) from The World Bank.
Total: 62% (USD$ 1682)	Setting: University hospital in Bangkok	
without complications: 49% (USD$ 1,329)	Sample and study design: The study included all 8,596 (94% insured, 6% uninsured) DM patients admitted in the hospital during 2006-2008. Retrospective data analysis.	
with myocardial infarction: 108% (USD$ 2,930)		
with congestive heart failure: 93% (USD$ 2523)		
with peripheral vascular disease: 116% (USD$ 3147)		
with ulcer: 106% (USD$ 2,876)		
with hemiplegia: 63% (USD$ 1,709)		
with moderate/severe renal disease: 90% (USD$ 2,442)		
Median cost of illness per year:	Study year: 2007-2008	[37] same study as [60]
With complications USD$ 480 (n = 148, IQR = 129-1552)	Setting: Waritchaphum hospital in Sakhon Nakhon province (North-East Thailand)	
Without complications USD$ 115 (n = 327, IQR = 74-286)	Sample and study design: 475 randomly selected diabetic patients. Micro-costing approach.	
With microvascular complications USD$ 641 (n = 59, IQR = 207-2,268)		
With macrovascular complications USD$ 367 (n = 11, IQR = 111-2,463)		
With micro- and macrovascular complications USD$ 666 (n = 11, IQR = 201-2,707)		
With microvascular complications and cataract USD$ 745 (n = 23, IQR = 376-1,358)		
Cataract USD$ 151 (n = 44, IQR = 94-587)		
Predicted cost per year of DM T2 (N = 186):	Study year: 2001	[52]
without complications USD$ 101 (BHT 4,037)	Setting: 30-bed public hospital in central Thailand	
with hypertension USD$ 117 (BHT 4,686)	Sample and study design: 186 diabetic patients. Retrospective prevalence-based cost of illness study. Multiple regression analysis was used to predict cost of DM for various types of complications.	
with hyperlipidaemia USD$ 144 (BHT 5,775)		
with diabetic foot USD$ 190 (BHT 7,603)		
with hyperglycaemia USD$ 209 (BHT 8,369)		
with hypoglycaemia USD$ 247 (BHT 9,860)		
with cerebrovascular accident USD$ 260 (BHT 10,418)		
with gangrene USD$ 336 (BHT 13,417)		
(40 THB = 1 USD$ at 2011 prices)		

Notes: SD: standard deviation, IQR: inter-quartile range.

There is a large variation in the estimates of the prevalence and costs of complications in Thailand. More studies at different levels of care and covering all the regions are needed to get a full picture of the prevalence and the cost of diabetes complications in the country.

## Conclusions

Based on the results of this study, the following priorities for the future management of diabetes in Thailand were identified. First, increasing screening of diabetes in high risk population and promoting annual screening of diabetes complications in all diabetic patients. Second, identifying and addressing factors affecting poor treatment outcomes in light of reducing the number of treated but not controlled patients and therefore reducing their likelihood of developing complications. Third, policy should specify clear targets and provide and use a monitoring framework to track progress.

Fourth, efforts are needed to further improve data availability. Up-to-date data on the medical and economic burden of diabetes representative at the national level and at least the regional level are essential to identify needs and monitor progress towards established targets. Priority areas for data collection include incidence of diabetes in children and adults, prevalence of GDM, cost of diabetes and its complications, and treatment compliance and outcomes at individual level. Availability of prevalence data is good due to the regular NHES, however, these surveys are only conducted every 5-7 years.

Fifth, promotion of a healthy lifestyle for prevention of diabetes through education and quality information delivered to the public.

Efforts to address these issues have already started in some areas of the country but not overall. In order to achieve this, a multisectoral effort including concerted policy actions from a variety of policy makers (beyond the ministry of public health and including other relevant ministries) and of public opinion leaders as well as interventions involving public and private delivery channels is required.

## Endnotes

^a^Study year is not specified but the publication data is 2000 so it is very likely to refer to the late 1990s.

^b^In 2004 prevalence in urban areas was higher for men but not for women.

## Abbreviations

BHT: Thai Bath; BMI: Body mass index; T2/1: Type 2/1; DALYs: Disability adjusted life years; DM: Diabetes mellitus; DT: Diabetes retinopathy; DN: Diabetes nephropathy; FPG: Fasting plasma glucose; GDM: Gestational diabetes mellitus; HbA1c: Glycaeted haemoglobin; MoPH: Ministry of Public Health; NCDs: Non-communicable diseases; NHES: National Health Examination Survey; NDDG: National Diabetes Data Group; OGTT: Oral glucose tolerance test; RRT: Renal replacement therapy; TDR: Thailand diabetes registry; UC: Universal coverage; USD: United States dollar

## Competing interests

This study was funded by NovoNordisk Switzerland. The funder had no involvement with the study design, data analysis, and paper writing. Both the authors declare no conflict of interest.

## Authors’ contributions

Both authors’ contributed to data acquisition, analysis and paper writing. CD conducted interviews with the MoPH, CD and AF conducted the systematic literature review, CD wrote the first draft, CD and AF redrafted and critically revised the paper and approved the final manuscript.

## References

[B1] Data [http://data.worldbank.org]. Accessed 01.03.2012

[B2] Bundhamcharoen K Odton P Phulkerd S Tangcharoensathien V Burden of disease in Thailand: changes in health gap between 1999 and 2004 BMC Public Health 2011 11 53 10.1186/1471-2458-11-53 21266087 PMC3037312

[B3] World Health Organization Non-communicable diseases country profile 2010 Geneva, Switzerland: WHO

[B4] World Health Organization Global Health Observatory 2010 Geneva, Switzerland: WHO

[B5] World Health Organization Country cooperation strategy at a glance 2010 Thailand

[B6] Tangcharoensathien V Swasdiworn W Jongudomusk P Srithamrongswat S Patcharanarumol W Prakongsai P Thammathataree J Universal coverage scheme in Thailand: Equity outcomes and future agendas to meet challenges Health systems financing The path to universal coverage World Health Report 2010 Bangkok, Thailand

[B7] Tangcharoensathien V Patcharanarumol W Vasavid C Prakongsai P Jongudomusk P Srithamrongswat S Thammathataree J Thailand Health Financing Review 2010 2010 Bangkok, Thailand: Thai working group on Observatory of Health Systems and Policy

[B8] Prakongsai P Limwattananon S Tangcharoensathien V Hanson K, Chernichovsky D The Equity impact of the universal coverage policy: lessons from Thailand Innovations in health system finance in developing and transitional economies 2009 21 London: Emerald Group Publishing Limited 57 81 19791699

[B9] Treerutkuarkul A Thailand: health care for all, at a price Bull World Health Organ 2010 88 2 84 85 20428360 10.2471/BLT.10.010210 PMC2814485

[B10] Sooksriwong C Yoongthong W Riewpaiboon A Pongcharoensuk P Thavorncharoensab M Chaikledkaew U Suwattanapreeda S Medicine Pricing, Availability and Affordability in Thailand Report of a survey conducted in Bangkok (Capital City), Phitsanulok (North), Suratthani (South), and Nakornrachaseema (Northeast) 2007 Bangkok, Thailand: The Office of Food and Drug Administration and The Ministry of Public Health

[B11] Aekplakorn W Chariyalertsak S Kessomboon P Sangthong R Inthawong R Putwatana P Taneepanichskul S Thai National Health Examination Survey IV Study Group Prevalence and management of diabetes and metabolic risk factors in Thai adults the Thai national health examination survey IV, 2009 Diabetes Care 2011 34 9 1980 1985 10.2337/dc11-0099 21816976 PMC3161276

[B12] Aekplakorn W Abbott-Klafter J Premgamone A Dhanamun B Chaikittiporn C Chongsuvivatwong V Suwanprapisa T Chaipornsupaisan W Tiptaradol S Lim SS Prevalence and management of diabetes and associated risk factors by regions of Thailand: Third National Health Examination Survey 2004 Diabetes Care 2007 30 8 2007 2012 10.2337/dc06-2319 17468342

[B13] Rawdaree P Ngarmukos C Deerochanawong C Suwanwalaikorn S Chetthakul T Krittiyawong S Benjasuratwong Y Bunnag P Kosachunhanun N Plengvidhya N Thailand diabetes registry (TDR) project: Clinical status and long term vascular complications in diabetic patients J Med Assoc Thai 2006 89 Suppl.1 S1 S9 17717877

[B14] Chuang L Tsai S Huang B Tai T on behalf of the Diabcare-Asia Study Group The status of diabetes control in Asia - a cross-sectional survey of 24 317 patients with diabetes mellitus in 1998 Diabet Med 2002 19 12 978 985 10.1046/j.1464-5491.2002.00833.x 12647837

[B15] Nitiyanant W Chetthakul T Sang-A-Kad P Therakiatkumjorn C Kunsuikmengrai K Yeo JP A survey study on diabetes management and complication status in primary care setting in Thailand J Med Assoc Thai 2007 90 1 65 71 17621735

[B16] Aekplakorn W Stolk R Neal B Suriyawongpaisal P Chongsuvivatwong V Cheepudomwit S Woodward M InterASIA Collaborative Group The Prevalence and Management of Diabetes in Thai Adults. The International Collaborative Study of Cardiovascular Disease in Asia Diabetes Care 2003 25 10 2758 2763 14514576 10.2337/diacare.26.10.2758

[B17] Jiamjarasrangsi W Aekplakorn W Incidence and predictors of type 2 diabetes among professional and office workers in Bangkok, Thailand J Med Assoc Thai 2005 88 12 1896 1904 16518992

[B18] Jiamjarasrangsi W Lohsoonthorn V Lertmaharit S Sangwatanaroj S Incidence and predictors of abnormal fasting plasma glucose among the university hospital employees in Thailand Diabetes Res Clin Pract 2008 79 2 343 349 10.1016/j.diabres.2007.09.008 17953998

[B19] Bhuripanyo K Ruangratanaamporn O Mahanonda N Leowattana W Sriratanasathaavorn C Chotinaiwattarakul C Kangkagate C Chaithiraphan S Impaired fasting glucose, diabetes mellitus and coronary risk factors J Med Assoc Thai 2000 83 Suppl.2 S146 S152 11194006

[B20] Likitmaskul S Angsusingha K Morris S Kiattisakthavee P Chaichanwatanakul K Tuchinda C Type 1 diabetes in Thai children aged 0-14 years J Med Assoc Thai 1999 82 8 826 832 10511793

[B21] Likitmaskul S Kiattisathavee P Chaichanwatanakul K Punnakanta L Angsusingha K Tuchinda C Increasing prevalence of type 2 diabetes mellitus in Thai children and adolescents associated with increasing prevalence of obesity J Pediatr Endocrinol Metab 2003 16 1 71 77 12585343 10.1515/jpem.2003.16.1.71

[B22] Panamonta O Laopaiboon M Tuchinda C Incidence of childhood type 1 (insulin dependent) diabetes mellitus in northeastern Thailand J Med Assoc Thai 2000 83 8 821 824 10998832

[B23] Patarakijvanich N Tuchinda C Incidence of diabetes mellitus type 1 in children of southern Thailand J Med Assoc Thai 2001 84 8 1071 1074 11758838

[B24] Tuchinda C Likitmaskul S Unachak K Panamonta O Patarakijavanich N Chetthakul T The epidemiology of type 1 diabetes in Thai children J Med Assoc Thai 2002 85 6 648 652 12322836

[B25] Unachak K Tuchinda C Incidence of type 1 diabetes in children under 15 years in northern Thailand, from 1991 to 1997 J Med Assoc Thai 2001 84 7 923 928 11759972

[B26] Panamonta O Thamjaroen J Panamonta M Panamonta N Suesirisawat C The rising incidence of type 1 diabetes in the northeastern part of Thailand J Med Assoc Thai 2011 94 12 1447 1450 22295730

[B27] Boriboonhirunsarn D Sunsaneevithayakul P Nuchangrid M Incidence of gestational diabetes mellitus diagnosed before 20 weeks of gestation J Med Assoc Thai 2004 87 9 1017 1021 15516000

[B28] Serirat S Deerochanawong C Sunthornthepvarakul T Jinayon P Gestational diabetes mellitus J Med Assoc Thai 1992 75 6 315 319 1487678

[B29] Sumeksri P Wongyai S Aimpun P Prevalence of gestational diabetes mellitus (GDM) in pregnant women aged 30 to 34 years old at Phramongkutklao Hospital J Med Assoc Thai 2006 89 Suppl 4 S94 S99 17726813

[B30] Chanprapaph P Sutjarit C Prevalence of gestational diabetes mellitus (GDM) in women screened by glucose challenge test (GCT) at Maharaj Nakorn Chiang Mai Hospital J Med Assoc Thai 2004 87 10 1141 1146 15560687

[B31] Weerakiet S Srisombut C Bunnag P Sangtong S Chuangsoongnoen N Rojanasakul A Prevalence of type 2 diabetes mellitus and impaired glucose tolerance in Asian women with polycystic ovary syndrome Int J Gynaecol Obstet 2001 75 2 177 184 10.1016/S0020-7292(01)00477-5 11684113

[B32] Ministry of Public Health Wibulpolprasert S Thailand Health Profile Report 2008-2010 2011 Bangkok, Thailand: The War Veterans Organizations of Thailand

[B33] Aekplakorn W Bunnag P Woodward M Sritara P Cheepudomwit S Yamwong S Yipintsoi T Rajatanavin R A risk score for predicting incident diabetes in the Thai population Diabetes Care 2006 29 8 1872 1877 10.2337/dc05-2141 16873795

[B34] Deerochanawong C Putiyanun C Wongsuryrat M Serirat S Jinayon P Comparison of National Diabetes Data Group and World Health Organization criteria for detecting gestational diabetes mellitus Diabetologia 1996 39 1070–1073 1070 1073 8877291 10.1007/BF00400656

[B35] Rao C Porapakkham Y Pattaraarchachai J Polpraser W Swampunyalert N Lopez A Verifying causes of death in Thailand: rationale and methods for empirical investigation Population Health Metrics 2010 8 11 148 154 10.1186/1478-7954-8-11 PMC2880956 20482758

[B36] Porapakkham Y Rao C Pattaraarchachai J Polprasert W Vos T Adair T Lopez A Estimated causes of death in Thailand, 2005: implications for health policy Population Health Metrics 2010 8 14 191 198 10.1186/1478-7954-8-14 PMC2885317 20482761

[B37] Chatterjee S Riewpaiboon A Piyauthakit P Riewpaiboon W Boupaijit K Panpuwong N Archavanuntagul V Cost of diabetes and its complications in Thailand: a complete picture of economic burden Health Soc Care Community 2011 19 3 289 298 10.1111/j.1365-2524.2010.00981.x 21276105

[B38] Chatterjee S Riewpaiboon A Piyauthakit P Riewpaiboon W Cost of informal care for diabetic patients in Thailand Prim Care Diabetes 2011 5 2 109 115 10.1016/j.pcd.2011.01.004 21334276

[B39] Ministry of Public Health Thailand healthy lifestyle strategic plan 2011-2020 2011 Bangkok, Thailand: The War Veterans Organizations of Thailand 58

[B40] Sranacharoenpong K Hanning R Diabetes prevention education program for community health care workers in Thailand J Community Health 2012 37 3 610 618 10.1007/s10900-011-9491-2 21971628

[B41] Swaddiwudhipong W Lerdlukanayonge P Chaovakiratipong C Nguntra P Mahasakpan P Koonchote S Boonmak C Screening assessment of the elderly in rural Thailand by a mobile unit Trans R Soc Trop Med Hyg 1996 90 3 223 227 10.1016/S0035-9203(96)90221-7 8758055

[B42] Swaddiwudhipong W Mahasakpan P Chaovakiratipong C Nguntra P Tatip Y Koonchote S Boonmak C Tharmaphornpilas P Screening assessment of persons 40-59 years of age in rural Thailand by a mobile health unit J Med Assoc Thai 1999 82 2 131 139 10087720

[B43] Sranacharoenpong K Hanning R Developing a diabetes prevention education programme for community health-care workers in Thailand: formative findings Prim Health Care Res Dev 2011 12 4 357 369 10.1017/S146342361100020X 22284949

[B44] Oba N McCaffrey R Choonhapran P Chutug P Rueangram S Development of a community participation program for diabetes mellitus prevention in a primary care unit, Thailand Nurs Health Sci 2011 13 3 352 359 21812881 10.1111/j.1442-2018.2011.00627.x

[B45] Thai Diabetes Treatment Guideline 2011 http://www.diabassocthai.org/index.php?option=com_content&view=article&id=12%3A-2551-&catid=2%3A2011-01-25-09-11-02&Itemid=6&lang=th. Accessed 08.04.2013.

[B46] WHO, IDF Definition and diagnosis of diabetes mellitus and intermediate hyperglycaemia: Report of a WHO/IDF consultation 2006 Geneva, Switzerland: World Health Organization/International Diabetes Federation 54

[B47] WHO, IDF Screening for type 2 diabetes: Report of a WHO and IDF meeting 2003 Geneva, Switzerland: World Health Organization/International Diabetes Federation 54

[B48] American Diabetes Association Executive Summary: Standards of Medical Care in Diabetes 2012 Diabetes Care 2012 35 1 10.2337/dc12-s004 PMC3632178 22187471

[B49] Sriwijitkamol A Moungngern Y Vannaseang S Assessment and prevalences of diabetic complications in 722 Thai type 2 diabetes patients J Med Assoc Thai 2011 94 Suppl 1 S168 S174 21721443

[B50] Chetthakul T Deerochanawong C Suwanwalaikorn S Kosachunhanun N Ngarmukos C Rawdaree P Krittiyawong S Leelawatana R Bunnag P Plengvidhya N Thailand diabetes registry project: prevalence of diabetic retinopathy and associated factors in type 2 diabetes mellitus J Med Assoc Thai 2006 89 Suppl 1 S27 S36 17715831

[B51] Narenpitak S Narenpitak A Prevalence of chronic kidney disease in type 2 diabetes in primary health care unit of Udon Thani province, Thailand J Med Assoc Thai 2008 91 10 1505 1513 18972892

[B52] Riewpaiboon A Pornlertwadee P Pongsawat K Diabetes cost model of a hospital in Thailand Value Health 2007 10 4 223 230 10.1111/j.1524-4733.2007.00172.x 17645676

[B53] Mayurasakorn K Somthip N Caengow S Chulkarat N Wanichsuwan M Glycemic control and microvascular complications among type 2 diabetes at primary care units J Med Assoc Thai 2009 92 8 1094 1101 19694336

[B54] Pratipanawatr T Rawdaree P Chetthakul T Bunnag P Ngarmukos C Benjasuratwong Y Leelawatana R Kosachunhanun N Plengvidhya N Deerochanawong C Thailand Diabetic Registry cohort: predicting death in Thai diabetic patients and causes of death J Med Assoc Thai 2010 93 Suppl 3 S12 S20 21299087

[B55] Pratipanawatr T Rawdaree P Chetthakul T Bunnag P Ngarmukos C Benjasuratwong Y Leelawatana R Kosachunhanun N Plengvidhya N Deerochanawong C Thailand diabetes registry project: current status of dyslipidemia in Thai diabetic patients J Med Assoc Thai 2006 89 Suppl 1 S60 S65 17715835

[B56] Ministry of Public Health Thailand health profile 2005-2007 2008 Bangkok, Thailand: The War Veterans Organizations of Thailand

[B57] Mobile Eye Care WDF 08-395 http://www.worlddiabetesfoundation.org/projects/thailand-wdf08-395. Accessed 08.04.2013

[B58] Sacks DA Hadden DR Maresh M Deerochanawong C Dyer AR Metzger BE Lowe LP Coustan DR Hod M Oats JJN Frequency of gestational diabetes mellitus at collaborating centers based on IADPSG consensus panel-Recommended criteria Diabetes Care 2012 3 526 528 22355019 10.2337/dc11-1641 PMC3322716

[B59] Upakdee N Pannarunothai S Medical charges for outpatients: a case study in three provinces using health insurance data J Health Sci 2003 12 775 787

[B60] Riewpaiboon A Chatterjee S Riewpaiboon W Piyauthakit P Disability and cost for diabetic patients at a public district hospital in Thailand Int J Pharm Pract 2011 19 2 84 93 10.1111/j.2042-7174.2010.00078.x 21385239

[B61] Goldhaber-Fiebert JD Li H Ratanawijitrasin S Vidyasagar S Wang XY Aljunid S Shah N Wang Z Hirunrassamee S Bairy KL Inpatient treatment of diabetic patients in Asia: evidence from India, China, Thailand and Malaysia Diabet Med 2010 27 1 101 108 10.1111/j.1464-5491.2009.02874.x 20121896

[B62] Riewpaiboon A Chatterjee S Piyauthakit P Cost analysis for efficient management: diabetes treatment at a public district hospital in Thailand Int J Pharm Pract 2011 19 5 342 349 10.1111/j.2042-7174.2011.00131.x 21899614

[B63] Chetthakul T Likitmaskul S Plengvidhya N Suwanwalaikorn S Kosachunhanun N Deerochanawong C Krittiyawong S Leelawatana R Benjasuratwong Y Bunnag P Thailand diabetes registry project: prevalence of diabetic retinopathy and associated factors in type 1 diabetes mellitus J Med Assoc Thai 2006 89 Suppl 1 S17 S26 17715830

[B64] Eppens MC Craig ME Jones TW Silink M Ong S Ping YJ Type 2 diabetes in youth from the Western Pacific region: glycaemic control, diabetes care and complications Curr Med Res Opin 2006 22 5 1013 1020 10.1185/030079906X104795 16709323

[B65] Jenchitr W Samaiporn S Lertmeemongkolchai P Chongwiriyanurak T Anujaree P Chayaboon D Pohikamjorn A Prevalence of diabetic retinopathy in relation to duration of diabetes mellitus in community hospitals of Lampang J Med Assoc Thai 2004 87 11 1321 1326 15825707

[B66] Leelawattana R Pratipanawatr T Bunnag P Kosachunhanun N Suwanwalaikorn S Krittiyawong S Chetthakul T Plengvidhya N Benjasuratwong Y Deerochanawong C Thailand diabetes registry project: prevalence of vascular complications in long-standing type 2 diabetes J Med Assoc Thai 2006 89 Suppl 1 S54 S59 17717878

[B67] Likitmaskul S Wacharasindhu S Rawdaree P Ngarmukos C Deerochanawong C Suwanwalaikorn S Chetthakul T Bunnag P Kosachunhanun N Plengvidhaya N Thailand diabetes registry project: type of diabetes, glycemic control and prevalence of microvascular complications in children and adolescents with diabetes J Med Assoc Thai 2006 89 Suppl 1 S10 S16 17715829

[B68] Krairittichai U Potisat S Jongsareejit A Sattaputh C Prevalence and risk factors of diabetic nephropathy among Thai patients with type 2 diabetes mellitus J Med Assoc Thai 2011 94 Suppl 2 S1 S5 21717870

[B69] Supapluksakul S Ruamviboonsuk P Chaowakul W The prevalence of diabetic retinopathy in Trang province determined by retinal photography and comprehensive eye examination J Med Assoc Thai 2008 91 5 716 722 18672638

[B70] Krittiyawong S Ngarmukos C Benjasuratwong Y Rawdaree P Leelawatana R Kosachunhanun N Plengvidhya N Deerochanawong C Suwanwalaikorn S Pratipanawatr T Thailand diabetes registry project: prevalence and risk factors associated with lower extremity amputation in Thai diabetics J Med Assoc Thai 2006 89 Suppl 1 S43 S48 17715833

[B71] Kosachunhanun N Benjasuratwong Y Mongkolsomlit S Rawdaree P Plengvidhya N Leelawatana R Bunnag P Pratipanawatr T Krittiyawong S Suwanwalaikorn S Thailand diabetes registry project: glycemic control in Thai type 2 diabetes and its relation to hypoglycemic agent usage J Med Assoc Thai 2006 89 Suppl 1 S66 S71 17715836

[B72] Worawongprapa O Glycemic control in diabetes with metabolic syndrome in community hospital J Med Assoc Thai 2008 91 5 641 647 18672625

[B73] Ngarmukos C Bunnag P Kosachunhanun N Krittiyawong S Leelawatana R Prathipanawatr T Plengvidhya N Benjasuratwong Y Suwanwalaikorn S Deerochanawong C Thailand diabetes registry project: prevalence, characteristics and treatment of patients with diabetic nephropathy J Med Assoc Thai 2006 89 Suppl 1 S37 S42 17715832

[B74] Kosachunhanun N Tongprasert S Rerkasem K Diabetic foot problems in tertiary care diabetic clinic in Thailand Int J Low Extrem Wounds 2012 11 2 124 127 10.1177/1534734612446967 22553278

